# 3D/3D Bamboo Charcoal/Bi_2_WO_6_ Bifunctional Photocatalyst for Degradation of Organic Pollutants and Efficient H_2_ Evolution Coupling with Furfuryl Alcohols Oxidation

**DOI:** 10.3390/molecules29112476

**Published:** 2024-05-24

**Authors:** Yanan Qu, Xiaolin Li, Kang Bu, Jiayi Zhang, Da Chen, Junhui Liang, Huayu Chen, Huafeng Li, Liqun Bai

**Affiliations:** 1 College of Chemistry and Materials Engineering, Zhejiang Agriculture and Forestry University, Hangzhou 311300, China; ququyanan117@stu.zafu.edu.cn (Y.Q.); lxlwood@163.com (X.L.); bk@stu.zafu.edu.cn (K.B.); 23604072004@stu.zafu.edu.cn (J.Z.); 2 College of Materials and Chemistry, China Jiliang University, Hangzhou 310018, China; dchen_80@hotmail.com (D.C.); nkljhyx@163.com (J.L.); hychen@cjlu.edu.cn (H.C.)

**Keywords:** biochar, bismuth tungstate, photocatalyst, degradation, furfuryl alcohol, H_2_ evolution

## Abstract

Photocatalysis is one of the most promising pathways to relieve the environmental contamination caused by the rapid development of modern technology. In this work, we demonstrate a green manufacturing process for the 3D/3D rod-shaped bamboo charcoal/Bi_2_WO_6_ photocatalyst (210BC-BWO) by controlled carbonization temperature. A series of morphology characterization and properties investigations (XRD, SEM, UV–vis DRS, transient photocurrent response, N_2_ absorption-desorption isotherms) indicate a 210BC-BWO photocatalyst with higher charge separation efficiency, larger surface area, and better adsorption capacity. The excellent photocatalytic performance was evaluated by degrading rhodamine B (RhB) (98.5%), tetracycline hydrochloride (TC-HCl) (77.1%), and H_2_ evolution (2833 μmol·g^−1^·h^−1^) coupled with furfuryl alcohol oxidation (3097 μmol·g^−1^·h^−1^) under visible light irradiation. In addition, the possible mechanisms for degradation of organic pollutants, H_2_ evolution, and furfuryl alcohol oxidation were schematically investigated, which make it possible to exert photocatalysis by increasing the active radical. This study shows that the combination of bamboo charcoal and bismuth tungstate can be a powerful photocatalyst that rationally combines H_2_ evolution coupled with furfuryl alcohol oxidation and degradation of pollutants.

## 1. Introduction

Environmental pollution and energy shortage is brought about by energy consumption and waste [1], and is also caused by discharge of pollutants containing a variety of organic compositions such as antibiotics [2], dyes [3], phenols [4], and heavy metals [5]. The use of photocatalytic degradation of pollutants and waste biomass to generate value-added products has attracted much attention, contributing significantly to low-cost renewable energy evolution, as well as solving environmental problems. Among various renewable green technologies, photocatalytic H_2_ evolution is one of the most promising candidates due to its low consumption, low environmental impact, and easy sustainable recycling [6]. Comparatively, photocatalytic biomass conversion has the advantages of high selectivity and high efficiency [7]. Abundant biomass and its derivatives (e.g., furfuryl alcohol (FFA), 5-hydroxymethylfurfural, etc.) arise from photosynthesis in natural plants, and further transform into high-value chemicals. They have attracted extensive interest in the field of energy conversion [8,9]. The results of this study showed that biomass has a stronger hole trapping capability compared to the usual sacrificial agents (methanol, lactic acid, and triethanolamine), and that the protons can consistently gain more electrons to produce H_2_ with excellent photocatalytic activity [10]. Yang et al. found that the production rates of H_2_ and furfural reached 471.35 µmol·g^−1^·h^−1^ and 206.19 µmol·g^−1^·h^−1^ on the Ni-Au/CN photocatalyst, respectively, which were 3.2 and 2.9 times higher than those on the Au/CN photocatalyst [11]. Hence, photocatalytic overall reaction of biomass and its derivatives is an ideal way to simultaneously produce H_2_ reduction products and value-added oxidation products.

In this regard, it is important to explore heterophase photocatalysts with high performance to realize such photocatalytic reactions. However, most of the traditional photocatalysts (TiO_2_, ZnO, etc.) have a wider band gap, high photogenerated electron–hole composite rate, and less solar absorption efficiency [12,13], which limits these photocatalysts’ practical applications. Consequently, constructing composites can improve the separation efficiency of photogenerated electron–hole pairs, and further improve the photocatalysts performance. Biochar is a carbonized material with the most potential, exhibiting a high carbon yield (27–45% wt%) [14], rich void structure [15], high specific surface area, high porosity [16], and excellent electrical conductivity [17]. The formation of biochar is affected by the feedstock type, hydrothermal temperature, reaction pressure and holding time regulation [18]. China is the world’s largest producer of bamboo resource possessor. The development and utilization of bamboo charcoal (BC) resources is of great significance and is in line with the current needs of environmental protection, energy saving, and low carbon economy [19]. Moreover, a remarkable feature of BC is that its pores become higher after high-temperature carbonization, which can effectively improve the separation efficiency of electron–hole pairs and greatly increase the specific surface area of BET [20]. Therefore, it is chosen as a favorable carrier for photocatalysts. The green photocatalyst obtained by the combination of BC and semiconductor materials is a hot topic in today’s research [21]. Zhang et al. [22] synthesized TiO_2_-loaded BC composites and found that TiO_2_/BC shows better photocatalytic performance than pure TiO_2_, which provided more active sites for the degradation of pollutants. However, bismuth tungstate (Bi_2_WO_6_) is one of the most promising semiconductor materials with narrow bandgap, low bandgap energy, high chemical stability, and high photoreactivity [23]. In addition, the different morphologies (nanosheets, bird’s nests, hollow spheres, octahedrons and bouquets) of Bi_2_WO_6_ were obtained by different synthetic methods, which show obvious differences in photocatalytic performance. The three-dimensional (3D) bouquet-shaped structure, Bi_2_WO_6_, possesses a larger specific surface. This key feature significantly enhances its opportunities for contact with organic pollutants [24]. Liang et al. [25] designed biomass carbon modified flower-like Bi_2_WO_6_ microspheres, in which the removal rate of TC-HCL by a Bi_2_WO_6_/C (6:1) sample was increased by 29.7% compared with that of pure Bi_2_WO_6_. Therefore, biochar has electron transport properties in the composite catalysts that favor the separation of charge carriers. Wu et al. [26] investigated the effects of different doping ratios of biochar on the performance of Bi_2_WO_6_. They revealed that the composite photocatalysts with a mass ratio of biochar to BWO of 5% were the most effective, with the kinetic rate of RhB solution degradation being 5.27 times higher than pure BWO. Up to now, there are no reports on photocatalytic H_2_ evolution coupled with waste biomass oxidation and simultaneous degradation of organic pollutants by simple assembly using BC as the carrier and Bi_2_WO_6_ as the semiconductor photocatalyst.

**Scheme 1:**
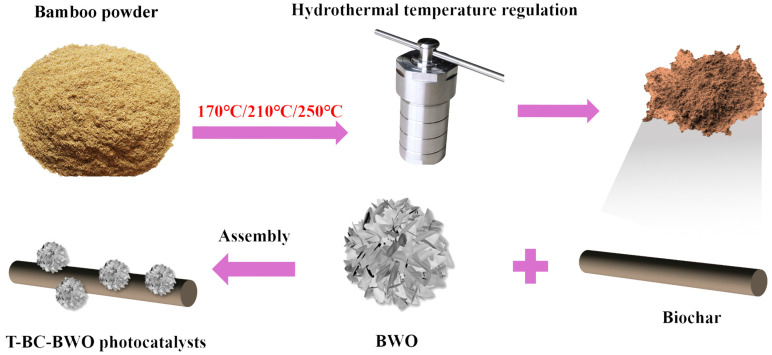
Schematic diagram of the T-BC-BWO photocatalysts.

Here, we have developed a novel, green and efficient 3D/3D rod/floral spherical T-BC-BWO photocatalyst based on 3D rod-shaped BC and 3D floral spherical BWO by simple hydrothermal method, which has excellent conductivity and high specific surface area. The photocatalytic composites were characterized by a series of morphological characterization and performance studies (XRD, SEM, UV–vis DRS, transient photocurrent response, and N_2_ absorption–adsorption isotherms). The excellent photocatalytic performance was evaluated by the degradation of RhB (98.5%) and TC-HCl (77.1%), and photocatalytic H_2_ evolution (2833 μmol·g^−1^·h^−1^) coupled with FFA oxidation (3097 μmol·g^−1^·h^−1^) under visible light irradiation. The results showed that the performance of 210BC-BWO was significantly improved compared with that of pure BWO. This study contributes to the construction of efficient rod/flower sphere composite photocatalysts for the removal of organic pollutants from water, while also realizing efficient photocatalytic H_2_ evolution coupled with FFA oxidation. It is a novel and meaningful strategy to kill two birds with one stone and has applications in both energy- and environment-related fields. Schematic diagram of the T-BC-BWO photocatalysts is shown in Scheme 1.

## 2. Results and Discussion

### 2.1. XRD Analysis

**Figure 1:**
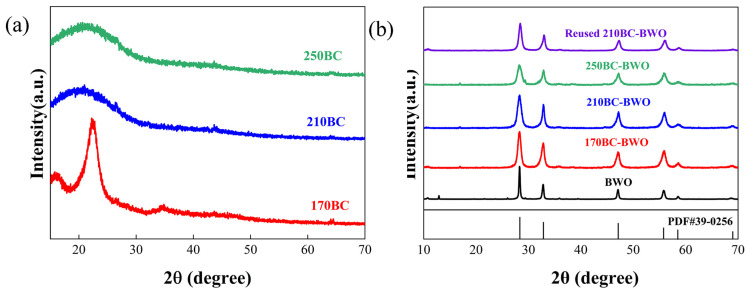
XRD patterns of (**a**) T-BC, (**b**) T-BC-BWO composite photocatalysts, and reused photocatalysts.

Figure 1a shows the XRD spectrum for three T-BC samples. As we know, cellulose has characteristic peaks at 16.3°, 22.3°, and 35°, corresponding to the crystalline structures of type II as (101), (002) and (040) [27]. The presence of characteristic peaks of cellulose in the diffraction pattern of 170BC indicated that the bamboo powder was not completely charred at 170 °C. The disappearance of the characteristic peaks of 210BC and 250BC indicate that the hydrothermal carbonization reaction destroys the crystalline structure of cellulose to form amorphous carbon at a higher temperature, and generates biomass carbon materials that possess high specific surface area, endowing the carbon material with high adsorption.

The XRD pattern of the T-BC-BWO composite sample is shown in Figure 1b. T-BC has no effect on the crystal structure of the composite photocatalysts, with peaks located at 28.2°, 32.7°, 47.1°, 55.8°, 58.6°, and 69.0°, corresponding to the (131), (200), (260), (133), (262), and (400) crystallographic planes of the orthorhombic BWOs [28]. For the following two reasons, the diffraction peaks of T-BC are not observed in all composites. Firstly, T-BC belongs to an amorphous structure and there is no obvious crystal diffraction information. Secondly, the diffraction peak intensity of T-BC is distinctly low compared with BWO.

### 2.2. SEM Analysis

**Figure 2:**
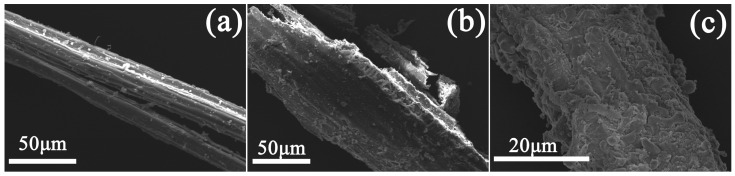
SEM images of BC with different carbonization temperature (T-BC), (**a**) 170BC, (**b**) 210BC, (**c**) 250BC.

The T-BC material has a fractured structure and a rougher surface with the increase in carbonization temperature (Figure 2). A pattern of 170BC (Figure 2a) maintains an intact rod structure, while 210BC (Figure 2b) basically maintains an overall rod structure but with localized fracture in the charcoal material. As for 250BC (Figure 2c), the BC underwent pyrolytic fracture under high-temperature conditions, and eventually formed a large accumulation of crumbly charcoal.

**Figure 3:**
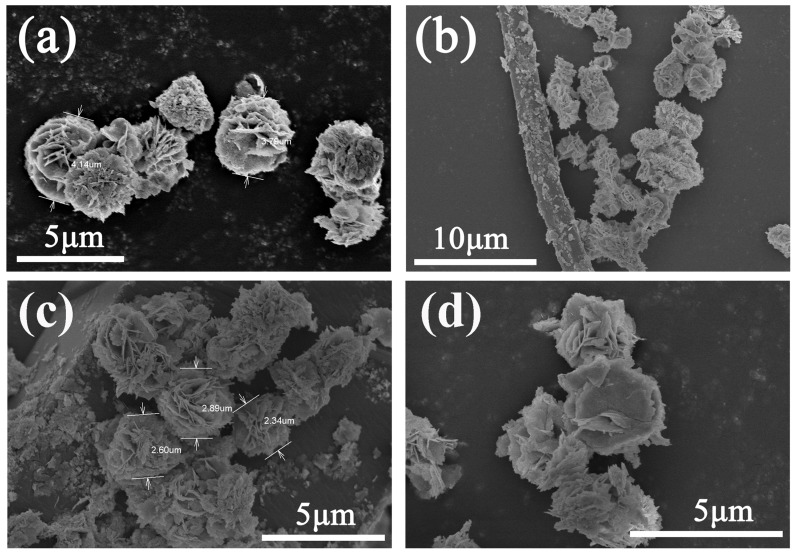
FESEM images of T-BC-BWO with different hydrothermal temperatures: (**a**) BWO; (**b**) 170BC-BWO; (**c**) 210BC-BWO; (**d**) 250BC-BWO.

Figure 3 displays Field Emission Scanning Electron Microscope (FESEM) images for the photocatalytic composites (170BC-BWO, 210BC-BWO, and 250BC-BWO). BWO composites show a 3D flower- and sphere-like structure with a diameter of approximately 4 μm (Figure 3a). The carbonization temperature has a large influence on the morphology of the T-BC-BWO composite photocatalyst. The morphology of 3D/3D 170BC-BWO consists of 3D rod-shaped 170BC and 3D flower-spherical BWO (Figure 3b). The 3D rod-shaped carbon material can improve the dispersion of catalysts in this 3D/3D structure. The morphology of 3D/3D 210BC-BWO showing the BWO flower ball is firmly fixed on the surface of 210BC, and the morphology and structure of the BWO flower bulbs were not changed by the incorporation of bamboo charcoal, but the size of the flower bulb decreased to 2 μm (Figure 3c). In contrast, 250BC-BWO is a composite of blob-shaped BWO and crumbled 250BC, the rod structure is crumbled to form a 3D/3D blob/blob-shaped 250BC-BWO (Figure 3d), which provides more uniform dispersion in treating organic pollutants. Therefore, the optimal hydrothermal temperature can be locked at 210 °C for bamboo charcoal composites, taking into account that the stabilized heterogeneous structure could exhibit excellent photocatalytic performance. In addition, as shown in the elemental mapping results C, O, Bi, and W elements are uniformly distributed in the hybrids, indicating the close coupling between BC and BWO, and the successful formation of 210BC-BWO composites (Figure S2).

### 2.3. BET Analysis

**Figure 4:**
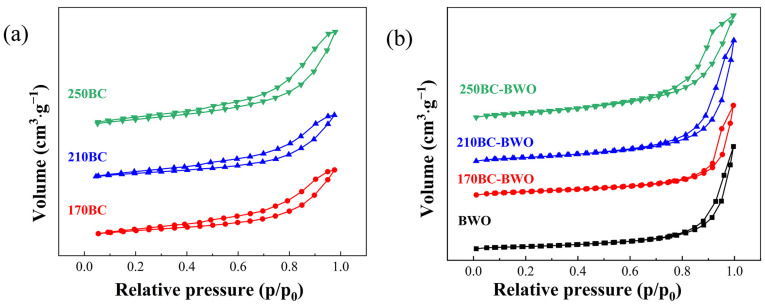
N_2_ absorption–desorption isotherms of (**a**) T-BC and (**b**) T-BC-BWO.

The N_2_ absorption–desorption isotherms for T-BC and T-BC-BWO are presented in Figure 4, and the associated pore information values can be found in Table 1. The pore sizes of the samples were almost distributed between 1 and 20 nm (Figure S4). All T-BC samples exhibit type III isotherms and H_4_ hysteresis loops (Figure 4a), which suggests that the increase in temperature did not change the pore structure of T-BC. All T-BC-BWO composites exhibit type III isotherms and H_3_ hysteresis loops and are similar to those of BWO (Figure 4b). This result indicates that the incorporation of BC under different hydrothermal temperatures does not alter the microporous and mesoporous structures. As expected, T-BC-BWO composites have a high specific surface area of 170BC-BWO (20.1 m^2^·g^−1^), 210BC-BWO (22.8 m^2^·g^−1^), and 250BC-BWO (29.8 m^2^·g^−1^). From the perspective of photocatalysis, a higher specific surface area tends to adsorb more target pollutants and form a dense target cover layer [29], which leads to a weakening of photocatalytic ability due to a reduction in visible light absorption performance. At the same time, their inappropriate band position and large overpotential for active radicals can also lead to slower photodegradation efficiency. This undesirable phenomenon been observed by the subsequent RhB degradation and H_2_ evolution coupled with FFA oxidation experiments [30].

### 2.4. UV–Vis Spectroscopy

**Figure 5:**
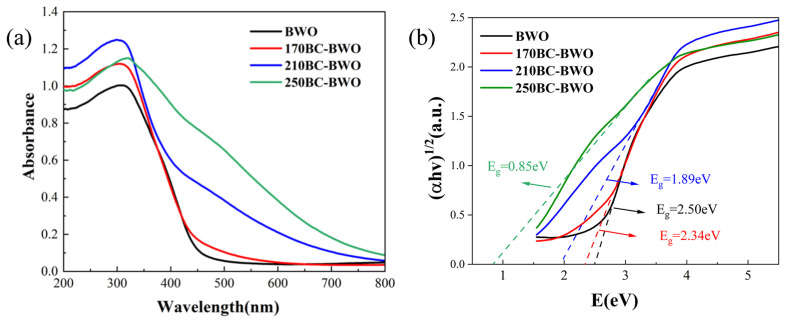
(**a**) UV–vis diffuse reflectance spectra; (**b**) forbidden bandwidths calculated according to the Tauc method for T-BC-BWO hybrid materials (E_g_).

The absorption edge of the T-BC-BWO composite material extends into the visible light region, indicating the potential for enhanced photocatalytic activity under visible light irradiation (Figure 5a). The E_g_ values for BWO and T-BC-BWO composites can be determined using Equation (1) in Supporting Information (Figure 5b), in which the E_g_ value (2.50 eV) of BWO is consistent with that listed in the relevant literature [31,32]. E_g_ values of the T-BC-BWO were reduced to 2.34 eV (170BC-BWO), 1.89 eV (210BC-BWO), and 0.85 eV (250BC-BWO), which may be attributed to the coupling effect between T-BC and BWO, reducing forbidden bandwidths [33].

### 2.5. Raman Spectra Analysis

**Figure 6:**
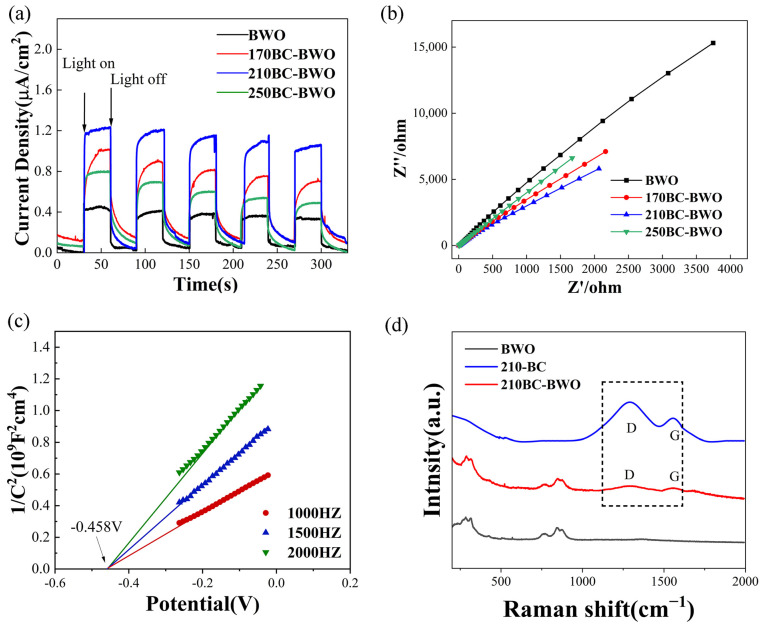
(**a**) Photocurrent density of BWO and T-BC-BWO photocatalysts; (**b**) EIS of BWO and T-BC-BWO photocatalysts; and (**c**) MS curves for 210BC-BWO; (**d**) Raman spectra of BC, BWO and 210BC-BWO samples.

The Raman spectra of BC, BWO, and 210BC-BWO samples are shown in Figure 6d. In the BC sample, carbon-specific D and G bands were observed at 1324 cm^−1^ and 1550 cm^−1^, respectively. This suggests that the hydrothermal carbonization reaction results in the formation of amorphous carbon. Since pure BWO does not contain carbon, no D and G peaks were observed. For 210BC-BWO, D and G bands were also observed at 1324 cm^−1^ and 1550 cm^−1^. Generally, the intensity of the G band is usually associated with the crystalline grains in the sample, while the intensity of the D band is typically related to structural defects. It can be demonstrated that its amorphous structure gives amorphous carbon a higher specific surface area and adsorption capacity. Therefore, the presence of amorphous structures endows the composite photocatalyst with a higher specific surface area and adsorption capacity.

### 2.6. Electrochemical Analysis

In order to investigate the effect of charge separation efficiency on catalytic activity, the transient photocurrent response and electrochemical impedance spectroscopy (EIS) of BWO and T-BC-BWO were tested using an electrochemical workstation (Figure 6). Under visible light irradiation, the charge separation of the catalyst was characterized by transient photocurrent responses (Figure 6a). Upon light excitation, a remarkably significant photocurrent was instantly generated, with the photocurrent intensity of all T-BC-BWO samples being noticeably enhanced, while the continuous cycles of photocurrent density for 210BC-BWO are higher than those of other samples, and the photocurrent response is increased by approximately three times compared to pure Bi_2_WO_6_. This is attributed to the conjugated π electron system of carbon increasing the electron export capability, further forming an observable photocurrent on the photoelectrode surface [34]. The presence of T-BC in the photocatalyst provides better carrier stability and carrier migration efficiency, thus facilitating the separation and transfer of photogenerated charges. The performance of interfacial charge separation efficiency can be clearly displayed by EIS Nengquis mapping (Figure 6b). The smaller the radius of the arc, the higher the efficiency of charge carrier transfer in the catalyst material [35]. All T-BC-BWO photocatalysts have smaller arc radii than BWO (BWO > 250BC-BWO > 170BC-BWO > 210BC-BWO), indicating that the participation of T-BC enhances the efficiency of the charge transfer at the complex interface between BWO and T-BC and inhibits the recombination of electron–hole pairs. Simultaneously, the Mott–Schottky (M-S) curves were measured to investigate the effect of introducing T-BC on the electrochemical properties. The 210-BC-BWO photocatalyst show positive slopes, thus this photocatalyst could be ascribed to an n-type semiconductor [36]. All flat band potentials of raw materials and composites were determined through analysis of the M-S curves combined in Equation (3) in Supporting Information. The flat band potentials (V_fb_) of the 210BC-BWO photocatalyst are −0.458 eV (vs. SCE) and −0.217 eV (vs. NHE).

### 2.7. Photocatalytic Performance for Degradation

**Figure 7:**
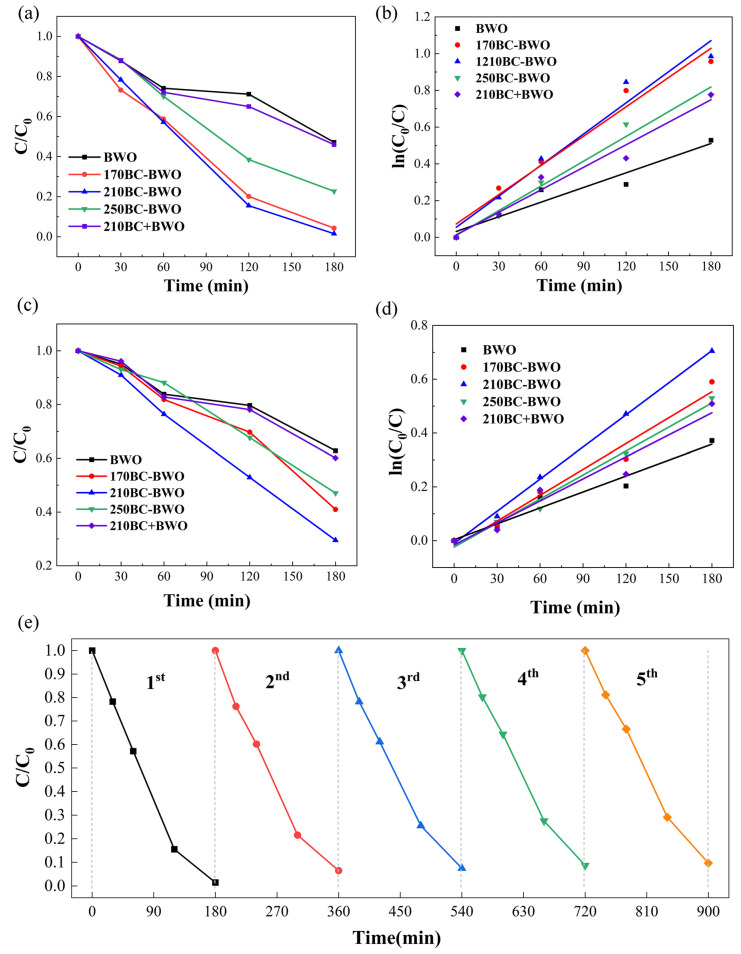
(**a**) Photocatalytic degradation curves of RhB (10 mg L^−1^) and (**b**) RhB (15 mg L^−1^) for different samples under visible light conditions; (**c**) first-order kinetics of RhB (10 mg L^−1^) and (**d**) (15 mg L^−1^) degradation under visible light; (**e**) visible photocatalytic stability diagram of 210BC-BWO photocatalyst.

The degradation kinetics of RhB are presented by BC, T-BC-BWO and 210BC+BWO photocatalyst under visible light (Figure 7). Generally speaking, the higher adsorption capacity creates more opportunities for the photocatalytic active site contact with the pollutant; however, 210BC-BWO with high charge separation efficiency and suitable band structure produces more active free radicals, which play a more important role for the RhB degradation (Figure S3). Based on the above analyses, 210BC-BWO displays good performance for the visible-light-driven photocatalytic degradation of organic dyes.

The first-order kinetic simulation plot (Figure 7c,d) has been proposed according to the photocatalytic degradation curves (Figure 7a,b) and Equation (2). The rate of degradation for RhB (10 mg L^−1^ and 15 mg L^−1^) solutions are in the following order: 210BC-BWO > 170BC-BWO > 250BC-BWO > 210BC + BWO > BWO. The above results demonstrate that the introduction of T-BC materials can significantly improve the photocatalytic performance of BWO (Figure 7).

**Figure 8:**
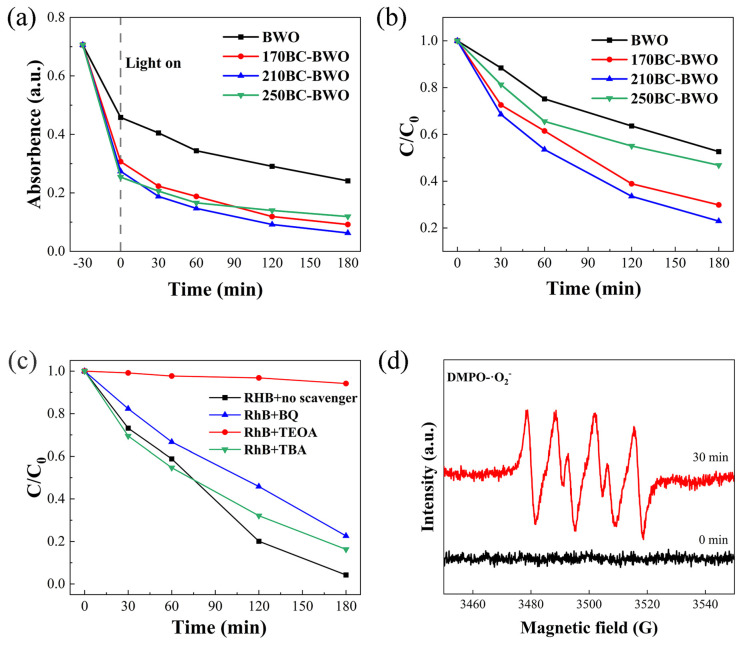
Photocatalytic degradation curves of (**a**) absorbance and (**b**) concentration for TC-HCl by T-BC-BWO photocatalyst under visible light conditions; (**c**) active species trapping experiment for the RhB degradation; (**d**) EPR spectra of 210BC-BWO with DMPO under visible light irradiation.

The stability of the 210BC-BWO photocatalyst was investigated by recycling experiments. Figure 7e shows that the degradation rate of RhB (10 mg L^−1^) in five rounds was 98.53%, 93.52%, 92.55%, 91.31%, and 90.32%, respectively, which proves that the photocatalyst 210BC-BWO has excellent cycling performance and stability. It can be observed that the characteristic diffraction peaks of the reused 210BC-BWO composite material are nearly unchanged. There are no disappearing peaks, nor are there any additional peaks, demonstrating that the prepared composite photocatalyst exhibits strong stability and can be reused repeatedly, as shown in Figure 1b. In the same pattern as that observed for the degradation of RhB, the initial concentration of the TC-HCl solution had a different degree of decrease after 30 min of dark adsorption, which was related to the adsorption capacity of T-BC-BWO (Figure 8). The dark adsorption results (Figure 8a) and photocatalytic efficiency (Figure 8b) together prove the feasibility and practicality of using T-BC-BWO photocatalysts for the removal of organic pollutants from wastewater [37,38,39].

In order to identify the main effect of active species in degradation organic pollutants, the various trapping agents been investigated in detail in the photocatalytic process (Figure 8c). The photocatalytic degradation of RhB with reduced activity was shown in presence of different trapping agents. Specifically, the addition of triethanolamine (TEOA) or benzoquinone (BQ) significantly decreased the photocatalytic degradation efficiency, while the degradation efficiency was only slightly inhibited by the participation of tert-butanol (TBA). Together, these results suggest that the main active substances in the photodegradation of organic pollutants were photogenerated holes (h^+^) and strong superoxide radicals (•O_2_^−^), rather than hydroxyl radicals (•OH). To further demonstrate the active species participating in the photocatalytic degradation of organic pollutants, in situ ESR spin trapping experiments were performed on 5,5-Dimethyl-1-pyrroline-N-oxide (DMPO) under visible light irradiation. The •O_2_^−^ signal was observed after light irradiation (Figure 8d), which indicates that •O_2_^−^ contributes during the degradation of organic pollutants.

### 2.8. Photocatalytic Performance for H_2_ Production Coupled with Selective Oxidation of Furfural Alcohol

**Figure 9:**
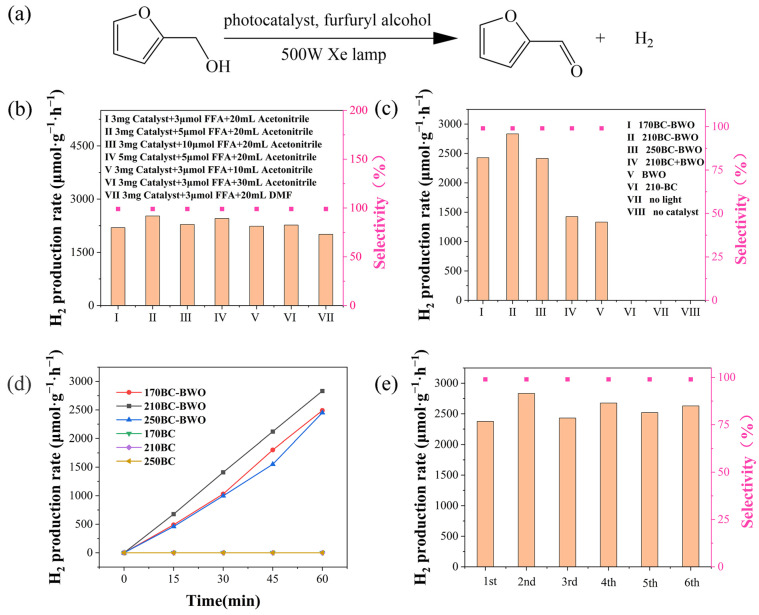
Photocatalytic performance of samples: (**a**) photocatalytic reaction; (**b**) condition exploration; (**c**) photocatalytic results for a series of control experiments; (**d**) time-dependent H_2_ evolution; (**e**) cyclic test of 210BC-BWO photocatalyst.

The photocatalytic performance of the prepared catalysts was evaluated by a photo-oxidation–reduction double reaction under simulated sunlight irradiation (Figure 9a). In this way, FFA can not only replace the traditional hole sacrificers with excellent H_2_ evolution, but also enable the production of value-added oxidation chemicals. Figure 9b summarizes the H_2_ yields of the same photocatalyst under different reaction conditions. The optimal reaction condition can be locked at 3 mg catalyst, 5 µmol FFA, and 20 mL acetonitrile. All the samples exhibited satisfactory H_2_ evolution capacity except for pure BC, while the H_2_-evolution capacity of T-BC-BWO was higher than that of pure BWO (332 μmol·g^−1^·h^−1^) (Figure 9c). Notably, the H_2_ yield of T-BC-BWO was further improved by regulating the hydrothermal temperature of composites. Among them, 210BC-BWO exhibits an ultra-high hydrogen generation rate of 2833 μmol·g^−1^·h^−1^, which is 2.12 times higher than that of the pristine BWO; this result further demonstrates the important role of the smaller forbidden band width. We deduce that the excellent H_2_ evolution ability of 210BC-BWO is due to the moderately narrowed forbidden band width, which favors the generation of more photogenerated electron–hole pairs (Figure S3). The linear relationship between H_2_ yield and time shows the stability of the catalyst (Figure 9d). Compared with T-BCs, all composite samples show satisfactory H_2_ generation capability, while 210BC-BWO has a higher H_2_ evolution capability than 170BC-BWO and 250BC-BWO, which further proves that 210BC, with its moderately narrow forbidden bandwidth, is favorable for the generation of more photogenerated electron–hole pairs. In addition, the cycling experiments were completed in the same system, Ar was introduced to remove the air and the residual H_2_ after each reaction, and the H_2_ yield was almost unchanged after six cycles, which further indicates that the binary composites have greater stability (Figure 9e). It is worth noting that the present study is in the upper-middle range of performance compared to the previous literature reports on furfural (FAL) photocatalytic oxidation (Table 2). In addition, the ratio of electrons to holes consumed in the redox reaction was calculated to be ca. 1.0, based on the co-production rates of H_2_ and FAL. This indicates that the dehydrogenation reaction proceeds at one stoichiometry, as shown in Equation (6) in Supporting Information. The photocatalytic H_2_ yields of T-BC-Bi_2_WO_6_ photocatalysts and the previously relevant FFA and H_2_ production coupled with aldehyde generation rates (including other composite photocatalysts and sacrificial agents) are shown in (Table 2).

### 2.9. Reaction Mechanism

Based on the relationship between the conduction band potential (CB), and the band gap energy (Eg) values, the valence band (VB) positions of the BWO and T-BC-BWO are calculated according to the empirical Equation (5) and are listed in Table S1 [47].

**Scheme 2:**
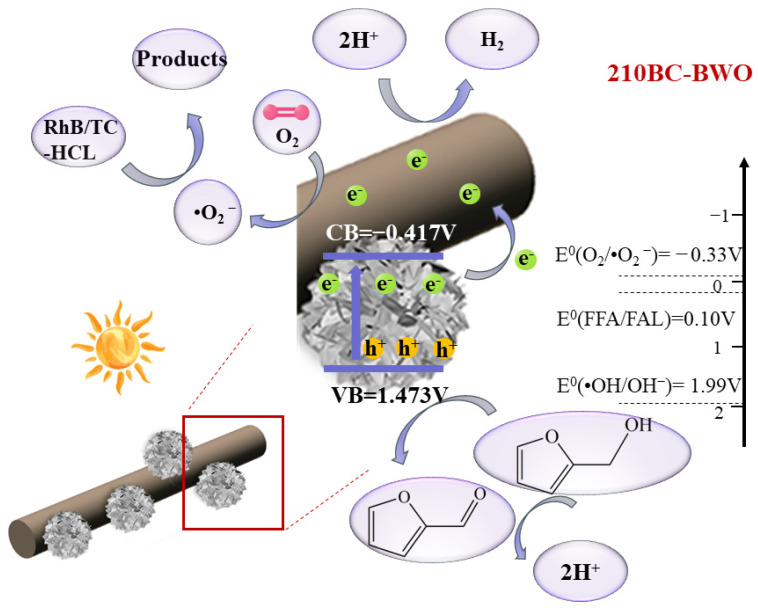
Diagrammatic depiction of the plausible photocatalytic mechanism involving the 210BC-BWO photocatalyst during the degradation of RhB/TC-HCL and H_2_ evolution coupled with FFA oxidation under visible light irradiation.

A possible mechanism for the photocatalytic degradation of organic pollutants and photocatalytic H_2_ evolution coupled with FFA oxidation was proposed according to the above result (Scheme 2). The CBs of 170BC-BWO and 210BC-BWO are more negative than the O_2_/•O_2_^−^ redox potential (−0.33 V vs. NHE) and their VBs are more positive than the FFA/FAL redox potential (0.10 V vs. NHE), which is feasible for the formation of active radicals in the reaction system for the degradation of organic pollutants [48,49] and H_2_ evolution coupled with FFA oxidation [41]. The VB values of 170BC-BWO and 210BC-BWO are not required for •OH/OH^−^ redox potential, which hinders the reaction between OH^−^ and h^+^ in producing •OH. Therefore, •OH does not contribute in the photocatalytic reaction system. The forbidden bandwidth value of 210BC-BWO is smaller than that of 170BC-BWO (Figure 7e), and the moderately narrowed forbidden bandwidth effectively improves the catalytic activity by light to produce more photogenerated electron–hole pairs. The CB and VB of 250BC-BWO are inadequate for the O_2_/•O_2_^−^ reduction potential and the •OH/OH^−^ oxidation potential; thus, 250BC-BWO shows the lowest photocatalytic performance. Apparently, the formation of e^−^, h^+^, and •O_2_^−^ active species play a key role in the degradation of pollutants and H_2_ evolution coupled with FFA oxidation by 210BC-BWO. Finally, the dual photo-oxidation–reduction reaction was carried out simultaneously, and the construction of the reaction system effectively prevented the accumulation of e^−^ and h^+^ on the surface of the catalyst with the excellent photocatalytic activity.

The above experimental data combined with the literature indicate the contribution of carbon materials to improving charge separation efficiency. The modification of BC in 210BC-BWO leads to a narrowing of the band gap, thereby facilitating the rapid generation of electron–hole pairs under visible light irradiation. Under visible light irradiation, the e^−^ of BWO are easily liberated from the confinement of electron–hole pairs (Equation (1)), and they swiftly transition from the VB state to the CB state within the band gap of BWO. Importantly, the incorporation of the electron acceptor BC into BWO is crucial, as it can rapidly shuttle the excited electrons from BWO’s CB state to the surface of BC (Equation (2)), thus preventing the recombination of photo-induced charge carriers in the composite system. Consequently, this leaves h^+^ at the VB position, which can directly oxidize adsorbed TC molecules (Equation (3)). Due to the close contact and reaction of a large number of e^−^ with oxygen molecules adsorbed on active sites of BC, more •O_2_^−^ is produced (Equation (4)). Finally, the formation of •O_2_^−^ strongly oxidizes organic pollutants (RhB, TC-HCl) into non-toxic small molecules (Equation (5)) [50,51]. Additionally, in the hydrogen production system, the strong oxidation hole from BWO promotes FFA to rapidly convert into FAL and release protons, and the photogenerated e^−^ from BC can readily reduce surface-adsorbed protons to generate H_2_ (Equations (1), (2), (4), (6) and (7)). Based on the above analysis, the stepwise mechanisms are proposed as follows:

Bi_2_WO_6_ + *hv* → h^+^(Bi_2_WO_6_) + e^−^(Bi_2_WO_6_) (1)

e^−^(Bi_2_WO_6_) → e^−^(biochar) (2)

h^+^(Bi_2_WO_6_) + RhB/TC-HCl → degraded products (3)

O_2_(biochar) + e^−^(biochar) → •O_2_^−^(biochar) (4)

•O_2_^−^(biochar) + RhB/TC-HCl → degraded products (5)

h^+^(Bi_2_WO_6_) + FFA → FAL + 2H^+^ (6)

2H^+^ + 2e^−^(biochar) → H_2_ (7)

## 3. Materials and Methods

### 3.1. Chemicals

Bismuth nitrate pentahydrate (Bi(NO_3_)_3_·5H_2_O) and sodium tungstate dihydrate (Na_2_WO_4_·2H_2_O) were purchased from Sinopharm Chemical Reagent Co. (Shanghai, China). Furfuryl alcohol, acetonitrile, RhB, and TC-HCl were purchased from Shanghai Maclean’s Biochemistry Co. (Shanghai, China). The other chemical reagents were of analytical grade and did not require further purification.

### 3.2. Preparation of T-BC-BWO Composite

Bamboo charcoal was synthesized by hydrothermal method using bamboo powder as carbon source. The method was as follows: 2.000 g of bamboo powder was washed with deionized water and ethanol, respectively, and subsequently mixed with 60 mL of deionized water, transferred to an autoclave lined with Teflon, and kept in a blast oven at a set temperature of T for 24 h. After the hydrothermal reaction, the product was rinsed 2–3 times with deionized water and anhydrous ethanol, filtered through a vacuum pump, and the solid product was dried in a vacuum oven at 60 °C. After thorough grinding in a mortar, the T-BC (T is 170 °C, 210 °C and 250 °C) powder was obtained. 

Typically, 1.5000 g of Bi(NO_3_)_3_·5H_2_O was dissolved in 10 mL of 1.6 mol L^−1^ nitric acid solution. After Bi(NO_3_)_3_·5H_2_O was completely dissolved, 10 mL of deionized water was added and the solution was diluted to 20 mL to obtain the precursor solution A. Meanwhile, 0.4728 g of Na_2_WO_4_·2H_2_O was dissolved in 20 mL of 2.0 mol L^−1^ NaOH solution by magnetic stirring to obtain the precursor solution B. After that, precursor solution B was added drop by drop under magnetic stirring to precursor solution A, and the pH of the resulting mixed solution was adjusted to 1 with an aqueous solution of 1.6 M HNO_3_ and 2 M NaOH. Subsequently, 0.09 g of T-BC was added to the above-mixed solution, and after magnetic stirring for 10 min and ultrasonic dispersing for 30 min, a homogeneous mixed solution was formed. This mixed solution was then transferred to a 100 mL Teflon-lined reactor and hydrothermally reacted at 170 °C for 12 h. After completion of the process, the reactor was removed and allowed to cool down naturally to room temperature. The reaction precipitate was collected by filtration through a Brinell funnel, washed several times and dried overnight. A brown colored T-BC-BWO composite photocatalyst powder was obtained. Depending on the preparation temperature of the added charcoal, the obtained products were labeled as 170BC-BWO, 210BC-BWO, and 250BC-BWO. In addition, 210BC was mechanically mixed with BWO, and recorded as 210BC + BWO.

### 3.3. Photocatalytic Experiments

The photocatalytic experiments were carried out in a 100 mL glass beaker containing a certain amount of the target solution and the prepared photocatalyst powder. The visible light source was a 500 W Xe lamp with a 420 nm cutoff filter placed vertically at a fixed distance of 15 cm from the glass beaker. Prior to the photocatalytic experiments, 0.05 g of the prepared photocatalyst powder was dispersed in 50 mL of RhB solution (10 mg L^−1^), the magnetic stirrer was turned on and kept in the dark for dark adsorption, with the aim of reaching adsorption equilibrium to exclude the effect of powder adsorption on the photocatalytic efficiency. During the photocatalytic experiments, approximately 4.0 mL of solution was withdrawn at predetermined time intervals with a pipette gun, fed into a medical syringe, filtered through a filter membrane (13 mm, 0.22 μm), and the resulting solution was transferred to a cuvette. The absorbance of RhB solution at the maximum spectral absorption peak (554 nm) was measured using a UV–vis spectrometer (Shanghai Jingke 722N UV-Vis Spectrometer, Shanghai, China) to assess the dye degradation process. The degradation process of TC-HCl was similar to that of RhB, except that the concentration of TC-HCl was 20 mg L^−1^ and the amount of photocatalyst was 0.05 g. The absorbance value of TC-HCl at 357 nm was measured and recorded by a double-beam UV spectrophotometer (Shanghai Jingke L5S, Shanghai, China) to evaluate the degradation rate of TC-HCl. The procedure for the reactive group burst experiment was similar to the photocatalytic experiment described above, except that 0.1 mM of a specific scavenger was added to the solution before the xenon lamp was turned on. The identification of h^+^, •OH and •O_2_^−^ was performed with triethanolamine (TEOA), tert-butanol (TBA) and p-benzoquinone (BQ), respectively.

Photocatalytic H_2_ evolution coupled with FFA oxidation experiments were conducted in a reactor with a quartz glass lid and a circulating water-cooling jacket. A total of 20 mL of 5 μmol FFA-acetonitrile solution and 3 mg of photocatalyst were introduced into the reactor. The reactor was then degassed under vacuum, energized with argon, and finally placed under a 500 W Xe lamp for 1 h. At the end of the reaction, the resulting solution was centrifuged and the supernatant was filtered through a syringe fitted with a 0.22 μm filter. The samples of H_2_ and furfural (FAL) were injected into gas chromatograph (GC9790 Plus, Fuli Analytical Instruments Corp, Hangzhou, China) equipped with flame ion detection.

## 4. Conclusions

In summary, a novel T-BC-BWO with 3D/3D rod/flower spherical morphology photocatalyst was synthesized by hydrothermal method. The removal efficiency of RhB and TC-HCl were 98.5% and 77.1%, and H_2_ evolution rate (2833 μmol·g^−1^·h^−1^) coupled with FFA oxidation (3097 μmol·g^−1^·h^−1^) by 210BC-BWO photocatalyst was conducted under visible light irradiation. The forbidden band width of the composite photocatalyst was reduced from 2.50 eV (BWO) to 1.89 eV (210BC-BWO), and the suitable VB and CB positions led to a substantial improvement in the catalytic performance. This study provides a bifunctional photocatalyst for the degradation of organic pollutants or the catalytic conversion of biomass FFA to H_2_ and FAL with high value-added chemicals and proposes a green and sustainable strategy for solving environmental pollution and energy shortages.

## Figures and Tables

**Table 1:** Pore properties of T-BC and T-BC-BWO samples.

Sample	BET Surface (m^2^·g^−1^)	Total Pore (cm^3^·g^−1^)	Average Pore Diameter ^a^ (nm)
170BC	50.8	0.12	3.7
210BC	52.1	0.21	3.8
250BC	65.8	0.17	3.9
BWO	16.8	0.07	17.8
170BC-BWO	20.1	0.07	15.1
210BC-BWO	22.8	0.10	18.4
250-BC-BWO	29.8	0.11	12.3

^a^ mesopore diameter determination using the BJH method.

**Table 2:** The photocatalytic H_2_ production and aldehyde generation rates (including other composite photocatalysts and sacrificial agents).

Photocatalyst	Weight (mg)	Light Source Details	Sacrificial Reagent	H_2_ Production Rate	Aldehyde Production Rate
210BC-Bi_2_WO_6_ (This work) *	3	500 W Xe	FFA	2833 µmol·g^−1^·h^−1^	3097 µmol·g^−1^·h^−1^
Ni-Au/CN [11]	10	200 W Xe	FFA	471.35 µmol·g^−1^·h^−1^	206.19 µmol·g^−1^·h^−1^
1%-MoS_2_-ZIS [40]	5	300 W Xe	FFA	68.8 µmol	78.8 µmol
20% RS/ZIS-Sv [41]	20	300 W Xe	FFA	10.8 mmol·g^−1^·h^−1^	9.2 mmol·g^−1^·h^−1^
ZnIn_2_S_4_/HPM [42]	5	300 W Xe	Benzyl alcohol	10.6 mmol·g^−1^·h^−1^	7.68 mmol·g^−1^·h^−1^
Bi/ZnIn_2_S_4_ [43]	15	300 W Xe	Benzyl alcohol	3658.8 µmol·g^−1^·h^−1^	1030.37 µmol·g^−1^·h^−1^
Bi_2_WO_6_/ZnIn_2_S_4_ [44]	20	150 W Xe	Methanol	131.8 µmol·g^−1^·h^−1^	N/A
ZnIn_2_S_4_/g-C_3_N_4_ [45]	5	300 W Xe	Triethanolamine	282 µmol·g^−1^·h^−1^	N/A
g-C_3_N_4_/Co-MOF (ZIF-67) [46]	5	300 W Xe	Lactic acid	302.7 µmol·g^−1^·h^−1^	N/A

* Reaction conditions: photocatalyst (3 mg), FFA (5 µmol), acetonitrile (20 mL), 500 W Xe lamp, room temperature, 1 h.

## Data Availability

Data are contained within the article.

## Supplementary Materials

The following supporting information can be downloaded at: https://www.mdpi.com/article/10.3390/molecules29112476/s1.

## References

[1] Tan H., Li J., He M., Li J., Zhi D., Qin F., Zhang C. (2021). Global Evolution of Research on Green Energy and Environmental Technologies: A Bibliometric Study. J. Environ. Manag..

[2] Wei Z., Liu J., Shangguan W. (2020). A Review on Photocatalysis in Antibiotic Wastewater: Pollutant Degradation and Hydrogen Production. Chin. J. Catal..

[3] Yang W., Ding K., Chen G., Wang H., Deng X. (2023). Synergistic Multisystem Photocatalytic Degradation of Anionic and Cationic Dyes Using Graphitic Phase Carbon Nitride. Molecules.

[4] Asencios Y.J.O., Lourenço V.S., Carvalho W.A. (2022). Removal of Phenol in Seawater by Heterogeneous Photocatalysis Using Activated Carbon Materials Modified with TiO_2_. Catal. Today.

[5] Gao X., Meng X. (2021). Photocatalysis for Heavy Metal Treatment: A Review. Processes.

[6] Balzani V., Bergamini G., Ceroni P. (2017). Photochemistry and Photocatalysis. Rendiconti Lincei.

[7] Wang D., Gong X.-Q. (2021). Function-Oriented Design of Robust Metal Cocatalyst for Photocatalytic Hydrogen Evolution on Metal/Titania Composites. Nat. Commun..

[8] Han G., Jin Y.-H., Burgess R.A., Dickenson N.E., Cao X.-M., Sun Y. (2017). Visible-Light-Driven Valorization of Biomass Intermediates Integrated with H_2_ Production Catalyzed by Ultrathin Ni/CdS Nanosheets. J. Am. Chem. Soc..

[9] Li C., Li J., Qin L., Yang P., Vlachos D.G. (2021). Recent Advances in the Photocatalytic Conversion of Biomass-Derived Furanic Compounds. ACS Catal..

[10] Li Y.-H., Zhang F., Chen Y., Li J.-Y., Xu Y.-J. (2020). Photoredox-Catalyzed Biomass Intermediate Conversion Integrated with H_2_ Production over Ti_3_C_2_T_x_/CdS Composites. Green Chem..

[11] Yang Q., Wang T., Han F., Zheng Z., Xing B., Li B. (2022). Bimetal-Modified g-C_3_N_4_ Photocatalyst for Promoting Hydrogen Production Coupled with Selective Oxidation of Biomass Derivative. J. Alloys Compd..

[12] Ma H., Shen J., Shi M., Lu X., Li Z., Long Y., Li N., Ye M. (2012). Significant Enhanced Performance for Rhodamine B, Phenol and Cr (VI) Removal by Bi_2_WO_6_Nancomposites via Reduced Graphene Oxide Modification. Appl. Catal. B Environ..

[13] Adhikari S., Kim D.-H. (2018). Synthesis of Bi_2_S_3_/Bi_2_WO_6_ Hierarchical Microstructures for Enhanced Visible Light Driven Photocatalytic Degradation and Photoelectrochemical Sensing of Ofloxacin. Chem. Eng. J..

[14] Zhang B., Heidari M., Regmi B., Salaudeen S., Arku P., Thimmannagari M., Dutta A. (2018). Hydrothermal Carbonization of Fruit Wastes: A Promising Technique for Generating Hydrochar. Energies.

[15] Wang Q. (2021). Peroxymonosulfate Activation by Tea Residue Biochar Loaded with Fe_3_O_4_ for the Degradation of Tetracycline Hydrochloride: Performance and Reaction Mechanism. RSC Adv..

[16] Carmona R.J., Velasco L.F., Hidalgo M.C., Navío J.A., Ania C.O. (2015). Boosting the Visible-Light Photoactivity of Bi_2_WO_6_ Using Acidic Carbon Additives. Appl. Catal. Gen..

[17] Ahmaruzzaman M. (2021). Biochar Based Nanocomposites for Photocatalytic Degradation of Emerging Organic Pollutants from Water and Wastewater. Mater. Res. Bull..

[18] Cui Z., Yang H., Wang B., Li R., Wang X. (2016). Effect of Experimental Parameters on the Hydrothermal Synthesis of Bi_2_WO_6_ Nanostructures. Nanoscale Res. Lett..

[19] Wang M., Huang Z.-H., Liu G., Kang F. (2011). Adsorption of Dimethyl Sulfide from Aqueous Solution by a Cost-Effective Bamboo Charcoal. J. Hazard. Mater..

[20] Yu X., Qin A., Liao L., Du R., Tian N., Huang S., Wei C. (2015). Removal of Organic Dyes by Nanostructure ZnO-Bamboo Charcoal Composites with Photocatalysis Function. Adv. Mater. Sci. Eng..

[21] Zhang J., Zhao D., Wang J., Yang L. (2009). Photocatalytic Oxidation of Dibenzothiophene Using TiO_2_/Bamboo Charcoal. J Mater. Sci..

[22] Wang W., Zhang J., Chen T., Sun J., Ma X., Wang Y., Wang J., Xie Z. (2020). Preparation of TiO_2_-Modified Biochar and Its Characteristics of Photo-Catalysis Degradation for Enrofloxacin. Sci. Rep..

[23] Orimolade B.O., Idris A.O., Feleni U., Mamba B. (2021). Recent Advances in Degradation of Pharmaceuticals Using Bi_2_WO_6_ Mediated Photocatalysis—A Comprehensive Review. Environ. Pollut..

[24] Wang W., Serp P., Kalck P., Faria J.L. (2005). Visible Light Photodegradation of Phenol on MWNT-TiO_2_ Composite Catalysts Prepared by a Modified Sol–Gel Method. J. Mol. Catal. Chem..

[25] Liang W., Pan J., Duan X., Tang H., Xu J., Tang G. (2020). Biomass Carbon Modified Flower-like Bi_2_WO_6_ Hierarchical Architecture with Improved Photocatalytic Performance. Ceram. Int..

[26] Wu G., Liu Q., Wang J., Zhang Y., Yu C., Bian H., Hegazy M., Han J., Xing W. (2022). Facile Fabrication of Bi_2_WO_6_/Biochar Composites with Enhanced Charge Carrier Separation for Photodecomposition of Dyes. Colloids Surf. Physicochem. Eng. Asp..

[27] Zhu J.-L., Chen S.-P., Lin W., Huang H.-D., Li Z.-M. (2023). Cellulose Mineralization with In-Situ Synthesized Amorphous Titanium Dioxide for Enhanced Adsorption and Auto-Accelerating Photocatalysis on Water Pollutant. Chem. Eng. J..

[28] Tian J., Sang Y., Yu G., Jiang H., Mu X., Liu H. (2013). A Bi_2_WO_6_-Based Hybrid Photocatalyst with Broad Spectrum Photocatalytic Properties under UV, Visible, and Near-Infrared Irradiation. Adv. Mater..

[29] Djellabi R., Yang B., Xiao K., Gong Y., Cao D., Sharif H.M.A., Zhao X., Zhu C., Zhang J. (2019). Unravelling the Mechanistic Role of TiOC Bonding Bridge at Titania/Lignocellulosic Biomass Interface for Cr (VI) Photoreduction under Visible Light. J. Colloid Interface Sci..

[30] Gholami P., Khataee A., Soltani R.D.C., Dinpazhoh L., Bhatnagar A. (2020). Photocatalytic Degradation of Gemifloxacin Antibiotic Using Zn-Co-LDH @ biochar Nanocomposite. J. Hazard. Mater..

[31] Wang T., Zhong S., Zou S., Jiang F., Feng L., Su X. (2017). Novel Bi_2_WO_6_-coupled Fe_3_O_4_ Magnetic Photocatalysts: Preparation, Characterization and Photodegradation of Tetracycline Hydrochloride. Photochem. Photobiol..

[32] Wang T., Liu S., Mao W., Bai Y., Chiang K., Shah K., Paz-Ferreiro J. (2020). Novel Bi_2_WO_6_ Loaded N-Biochar Composites with Enhanced Photocatalytic Degradation of Rhodamine B and Cr (VI). J. Hazard. Mater..

[33] Liu Q., Shen J., Yu X., Yang X., Liu W., Yang J., Tang H., Xu H., Li H., Li Y. (2019). Unveiling the Origin of Boosted Photocatalytic Hydrogen Evolution in Simultaneously (S, P, O)-Codoped and Exfoliated Ultrathin g-C_3_N_4_ Nanosheets. Appl. Catal. B Environ..

[34] Chung H.Y., Toe C.Y., Chen W., Wen X., Wong R.J., Amal R., Abdi F.F., Ng Y.H. (2021). Manipulating the Fate of Charge Carriers with Tungsten Concentration: Enhancing Photoelectrochemical Water Oxidation of Bi_2_WO_6_. Small.

[35] Wang J. (2020). Rapid Toxicity Elimination of Organic Pollutants by the Photocatalysis of Environment-Friendly and Magnetically Recoverable Step-Scheme SnFe_2_O_4_/ZnFe_2_O_4_ Nano-Heterojunctions. Chem. Eng. J..

[36] Xu T., Zhang L., Cheng H., Zhu Y. (2011). Significantly Enhanced Photocatalytic Performance of ZnO via Graphene Hybridization and the Mechanism Study. Appl. Catal. B Environ..

[37] Zhang S., Khan I., Qin X., Qi K., Liu Y., Bai S. (2020). Construction of 1D Ag-AgBr/AlOOH Plasmonic Photocatalyst for Degradation of Tetracycline Hydrochloride. Front. Chem..

[38] Lwin H.M., Zhan W., Jia F., Song S. (2022). Microwave-Assisted Hydrothermal Synthesis of MoS_2_-Ag_3_PO_4_ Nanocomposites as Visible Light Photocatalyst for the Degradation of Tetracycline Hydrochloride. Environ. Technol..

[39] Wu Y.X., Zeng G., Jiang L., Zhong H., Xie Y., Wang H., Chen X., Wang H. (2018). Highly Efficient Photocatalytic Activity and Mechanism of Yb^3+^/Tm^3+^ Codoped In_2_S_3_ from Ultraviolet to near Infrared Light towards Chromium (VI) Reduction and Rhodamine B Oxydative Degradation. Appl. Catal. B Environ..

[40] Tan C.-L., Qi M.-Y., Tang Z.-R., Xu Y.-J. (2021). Cocatalyst Decorated ZnIn_2_S_4_ Composites for Cooperative Alcohol Conversion and H_2_ Evolution. Appl. Catal. B Environ..

[41] Hu J., Li X., Qu J., Yang X., Cai Y., Yang T., Yang F., Li C.M. (2023). Bifunctional Honeycomb Hierarchical Structured 3D/3D ReS_2_/ ZnIn_2_S_4_-Sv Heterojunction for Efficient Photocatalytic H_2_-Evolution Integrated with Biomass Oxidation. Chem. Eng. J..

[42] Xing F., Zeng R., Cheng C., Liu Q., Huang C. (2022). POM-Incorporated ZnIn_2_S_4_ Z-Scheme Dual-Functional Photocatalysts for Cooperative Benzyl Alcohol Oxidation and H_2_ Evolution in Aqueous Solution. Appl. Catal. B Environ..

[43] He Y., Liu Y., Zhang Z., Wang X., Li C., Chen X.-B., Shi Z., Feng S. (2023). Atomically Dispersed Bismuth on ZnIn_2_S_4_ Dual-Functional Photocatalyst for Photocatalytic Hydrogen Production Coupled with Oxidation of Aromatic Alcohols to Aldehydes. Appl. Surf. Sci..

[44] Chachvalvutikul A., Luangwanta T., Pattisson S., Hutchings G.J., Kaowphong S. (2021). Enhanced Photocatalytic Degradation of Organic Pollutants and Hydrogen Production by a Visible Light–Responsive Bi_2_WO_6_/ZnIn_2_S_4_ Heterojunction. Appl. Surf. Sci..

[45] Zhang Z., Liu K., Feng Z., Bao Y., Dong B. (2016). Hierarchical Sheet-on-Sheet ZnIn_2_S_4_/g-C_3_N_4_ Heterostructure with Highly Efficient Photocatalytic H_2_ Production Based on Photoinduced Interfacial Charge Transfer. Sci. Rep..

[46] Devarayapalli K.C., Vattikuti S.V.P., Sreekanth T.V.M., Yoo K.S., Nagajyothi P.C., Shim J. (2020). Hydrogen Production and Photocatalytic Activity of g-C_3_N_4_/Co-MOF (ZIF-67) Nanocomposite under Visible Light Irradiation. Appl. Organomet. Chem..

[47] Liang Q., Zhang M., Liu C., Xu S., Li Z. (2016). Sulfur-Doped Graphitic Carbon Nitride Decorated with Zinc Phthalocyanines towards Highly Stable and Efficient Photocatalysis. Appl. Catal. Gen..

[48] Zhong X., Liu Y., Hou T., Zhu Y., Hu B. (2022). Effect of Bi_2_WO_6_ Nanoflowers on the U(VI) Removal from Water: Roles of Adsorption and Photoreduction. J. Environ. Chem. Eng..

[49] Wan J., Xue P., Wang R., Liu L., Liu E., Bai X., Fan J., Hu X. (2019). Synergistic Effects in Simultaneous Photocatalytic Removal of Cr (VI) and Tetracycline Hydrochloride by Z-Scheme Co_3_O_4_/Ag/Bi_2_WO_6_ Heterojunction. Appl. Surf. Sci..

[50] Tang G., Zhang F., Huo P., Zulfiqarc S., Xu J., Yan Y., Tang H. (2019). Constructing Novel Visible-Light-Driven Ternary Photocatalyst of AgBr Nanoparticles Decorated 2D/2D Heterojunction of g-C_3_N_4_/BiOBr Nanosheets with Remarkably Enhanced Photocatalytic Activity for Water-Treatment. Ceram. Int..

[51] Wu X., Wang X., Wang F., Yu H. (2019). Soluble g-C_3_N_4_ Nanosheets: Facile Synthesis and Application in Photocatalytic Hydrogen Evolution. Appl. Catal. B Environ..

