# Supplementary material for: 3D/3D Bamboo Charcoal/Bi2WO6 Bifunctional Photocatalyst for Degradation of Organic Pollutants and Efficient H2 Evolution Coupling with Furfuryl Alcohols Oxidation
Source: Molecules. 2024 May 24;29(11):2476. doi: 10.3390/molecules29112476 (PMC11174113; doi:10.3390/molecules29112476)
Supplement: Supplementary file 1 [file molecules-29-02476-s001.zip › molecules-3008190-supplementary.pdf]

Supporting Information

3D/3D Bamboo Charcoal/Bi_2WO_6 Bifunctional Photocatalyst for Degradation of Organic Pollutants and Efficient H_2 Evolution Coupling with Furfuryl Alcohols Oxidation

Yanan Qu [1], Xiaolin Li [1], Kang Bu [1], Jiayi Zhang [1], Da Chen [2], Junhui Liang [2], Huayu Chen [2], Huafeng Li [1,*] and Liqun Bai [1,*]

[1] College of Chemistry and Materials Engineering, Zhejiang Agriculture and Forestry University, Hangzhou 311300, China; ququyanan117@stu.zafu.edu.cn (Y.Q.); lxlwood@163.com (X.L.); bk@stu.zafu.edu.cn (K.B.); 23604072004@stu.zafu.edu.cn (J.Z.)

[2] College of Materials and Chemistry, China Jiliang University, Hangzhou 310018, China; dchen_80@hotmail.com (D.C.); nkljhyx@163.com (J.L.); hychen@cjlu.edu.cn (H.C.)

* Correspondence: lihuafeng@zafu.edu.cn (H.L.); bailiqun78@163.com (L.B.)

This files contains following contents:

Figure S1. EDS-mapping pattern of 210BC-BWO.

Figure S2. EDS elemental mapping (C, O, W, and Bi) in the 210BC-BWO composite and FESEM images of 210BC-BWO.

Figure S3. Schematic illustration of specific band positions of BWO and T-BC-BWO.

Figure S4. Pore size distribution of (**a**) T-BC and (**b**) T-BC-BWO

Table S1. Flat-band potentials, VB and CB positions and forbidden band widths of photocatalysts.

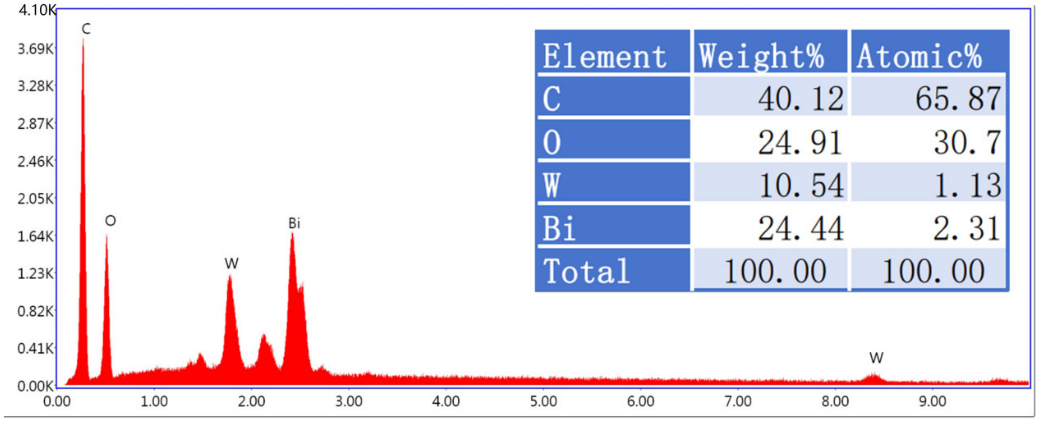

Figure S1. EDS-mapping pattern of 210BC-BWO.

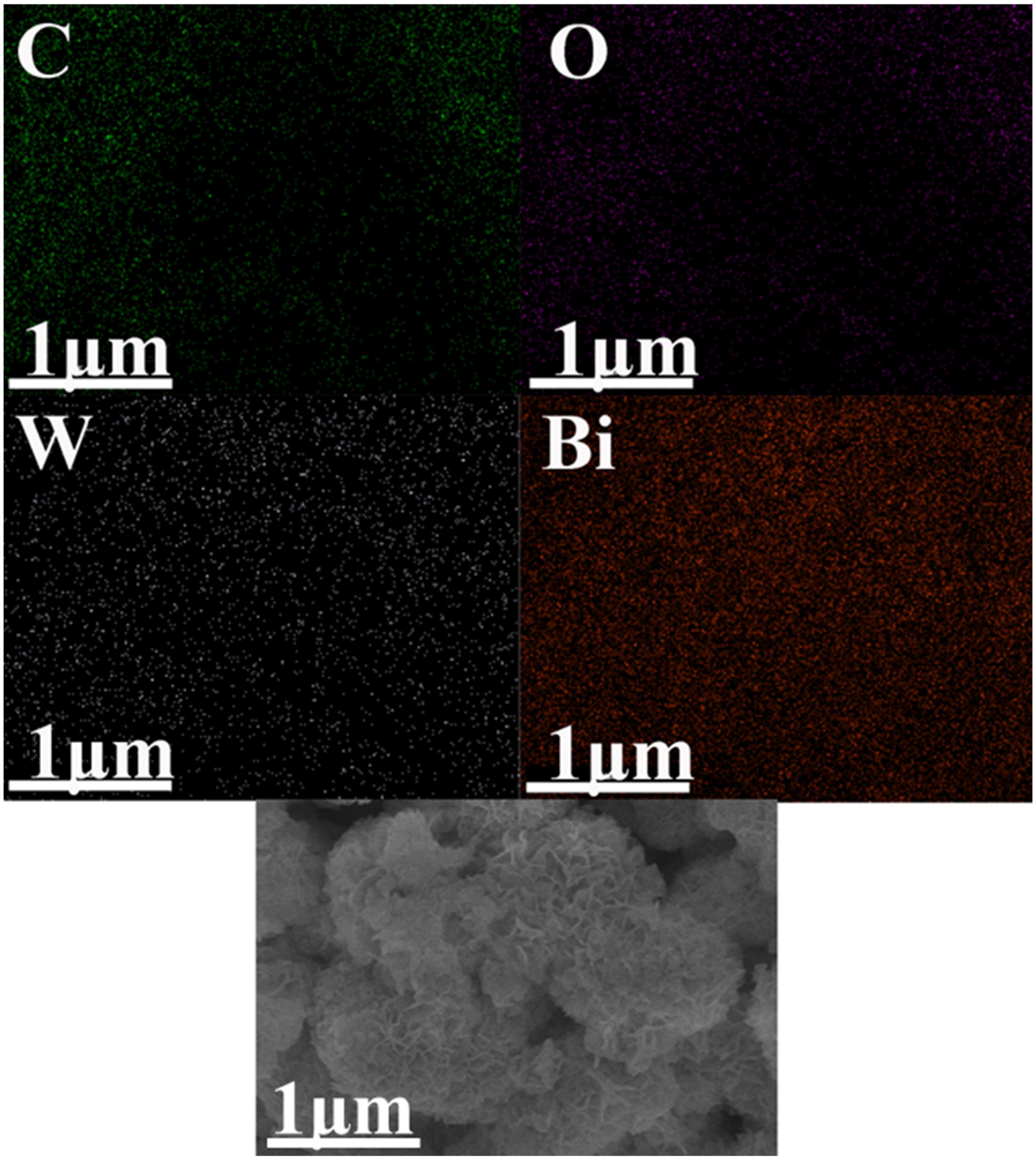

Figure S2. EDS elemental mapping (C, O, W, and Bi) in the 210BC-BWO composite and FESEM images of 210BC-BWO.

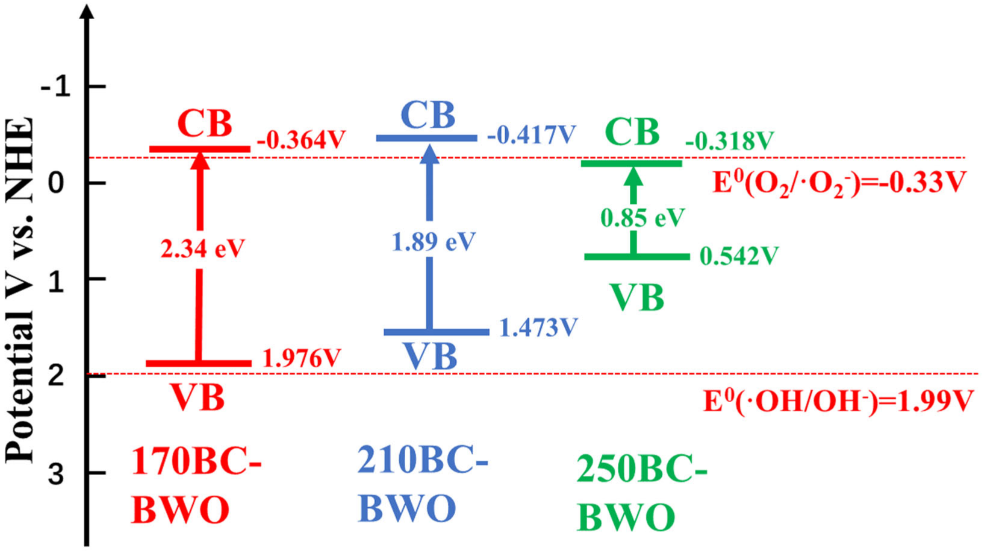

Figure S3. Schematic illustration of specific band positions of BWO and T-BC-BWO.

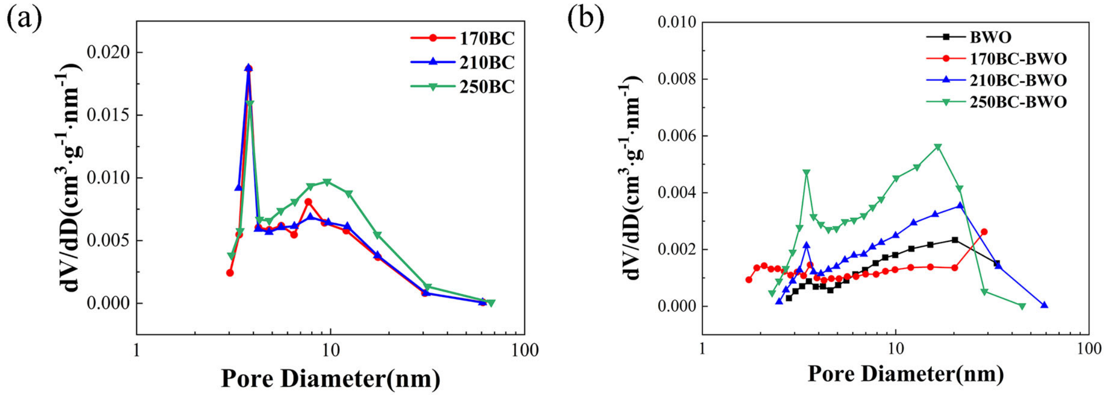

Figure S4. Pore size distribution of (**a**) T-BC and (**b**) T-BC-BWO

Table S1. Flat-band potentials, VB and CB positions and forbidden band widths of photocatalysts.

sample	V_{fb}（vs.NHE）	E_{CB}	E_{VB}	E_g
BWO	-0.200	-0.400	2.100	2.50
170BC-BWO	-0.164	-0.364	1.976	2.34
210BC-BWO	-0.217	-0.417	1.473	1.89
250BC-BWO	-0.118	-0.318	0.542	0.85

Characterization

Morphological characteristics of the prepared samples were examined using a Hitachi SU8010 field-emission scanning electron microscope attached with an energy-dispersive X-ray spectrometer. The crystal phases of the prepared samples were analyzed by X-ray diffraction (XRD) using a Rigaku Ultima Xray diffractometer with Cu as the radiation source. Morphological features of the prepared samples were examined using FEI Quanta scanning electron microscope (SEM). Fourier transform infrared (FT-IR) spectra were recorded by an infrared spectrometer (Shimadzu IR Prestige-21, Japan) to analyze the functional groups of the samples at wavelengths from 4000 to 400 cm^{-1} with a resolution better than 0.09 cm^{-1}. The nitrogen adsorption isotherms and special surface areas were obtained using an N_2 sorption analyzer (Micromeritics APSP 2460, USA). The special surface areas were calculated using the Brunauer-Emmett-Teller (BET) model, and the average pore diameters were calculated using the Barrett-Joyner-Halenda (BJH) method. Diffuse reflectance spectroscopy (DRS) was performed by a Shimadzu UV-3600i Plus UV/vis/NIR spectrophotometer using $BaSO_4$ as the reflectance standard. The electron spin resonance (ESR) result was examined by using a Bruker EMX plus-6/1 spectrometer using a spin-trap reagent (i.e.,5,5-dimethyl-L-pyrroline *N*-oxide (DMPO)) in the visible-light mode.

Photoelectrochemical experiments

To prepare working photoelectrodes for photoelectrochemical characterization, the FTO conductive glass was cleaned sequentially by ultrasonic cleaning in deionized water, soapy water, acetone and anhydrous ethanol for 10 min. The working photoelectrode is prepared by scraping a slurry on FTO conductive glass. The slurry was made by mixing the obtained photocatalyst powder and polyethylene glycol 6000 (PEG6000) in the ratio of 90:10 by weight, and then dried in a vacuum oven at 170°C for 4 hours. The active area of the photoelectrode was controlled to be 1.0 cm × 1.0 cm, and the visible light source was a 500 W Xe lamp fitted with a cutoff filter (λ ≥ 420 nm).

Photocurrent response (i-t), electrochemical impedance spectroscopy (EIS) and Mott-Schottky (M-S) curves were carried out on an electrochemical workstation (CHI660C) using a standard three-electrode system in a 0.5 mol L^{-1} Na_2SO_4 (pH=6.8) solution using a working photoelectrode, saturated calomel electrode (SCE) as the reference electrode, and platinum wire as the counter electrode, respectively.

Data analysis

The Kubelka-Munk equation (Eq. (1)) can be used to calculate the band gap energy of the prepared samples.

$$(\alpha hv)^{n} = A\ (hv - E_{g}) \quad (1)$$

α is the absorption coefficient, h is Planck's constant, v is the frequency of light, E_g is the band gap energy, and A is a constant. For direct or indirect transition semiconductors, n is 1/2 or 2, respectively. Thus, the band gap energy (Eg) of the obtained samples can be estimated from plots of $(\alpha hv)^2$ or $(\alpha hv)^{1/2}$ versus the energy of the directly and indirectly transformed photons (hv), respectively.

The photocatalytic reaction was fitted by the proposed first-order model and the rate constants were calculated by Eq. (2).

$$\ln(C_0/C) = kt \quad (2)$$

C_0 is the initial concentration of the solution before degradation, C is the remaining concentration of the solution after photodegradation at time t, k is the photocatalytic degradation rate constant, and t is the photocatalytic degradation time.

The flat band potential of a semiconductor material can be calculated from the Mott-Schottky curve using the following equation (Equation (3)).

$$1/C^2 = 2(E-V_{fb}-k_BT/e)/A^2e\varepsilon\varepsilon_0N_\mathrm{A} \qquad (3)$$

Inside，C denotes the interfacial capacitance, A denotes the electrode surface area, e denotes the electron charge, ε denotes the relative permittivity of the semiconductor, ε_0 denotes the vacuum permittivity, N_A denotes the carrier concentration, E denotes the applied potential, V_{fb} denotes the flat-carbon-potential of the semiconductor, k_B denotes the Boltzmann's constant, and T denotes the absolute temperature.

The flat-band potential obtained by Eq. (3) can be converted to an electrode potential relative to the general hydrogen electrode (NHE) using Eq. (4).

$$V_\mathrm{NHE} = V_\mathrm{SCE} + V^0_\mathrm{SCE} \qquad (4)$$

V_{NHE} is the converted potential, V^0_SCE (0.241 V, 298 K) and V_{SCE} are the standard potential and the experimental potential measured against SCE.

Empirical formula for band gap calculation：

$$E_g = E_{VB} - E_{CB} \qquad (5)$$

E_g is the photocatalyst band gap; E_{VB} is the VB potential; E_{CB} is the CB potential.

The ratio of electrons and holes, $R(e^- /h^+)$ consumed in the reaction was calculated using the equation:

$$R(e^- /h^+) = n_{H_2}/n_{FAL} \qquad (6)$$

The selectivity of producing furfural was calculated according to the following formula:

$$Selectivity\ (\%) = (C_{FFA}/\Sigma C_{products}) \times 100\% \qquad (7)$$
